# Revealing the Active State of a Cu/ZnO:Al Catalyst During Reverse Water–Gas Shift Reaction in an Operando Microwave Absorption Study

**DOI:** 10.1002/anie.202504280

**Published:** 2025-09-30

**Authors:** Zohreh Asadi, Clara Patricia Marshall, Annette Trunschke, Thomas Risse

**Affiliations:** ^1^ Institute of Chemistry and Biochemistry Freie Universität Berlin Arnimallee 22 14195 Berlin Germany; ^2^ Department of Inorganic Chemistry Fritz‐Haber‐Institut der Max‐Planck‐Gesellschaft Faradayweg 4–6 14195 Berlin Germany

**Keywords:** Cu/ZnO:Al catalyst, Operando EPR spectroscopy, Operando MCPT, Reaction mechanism, Reverse water—gas shift reaction

## Abstract

The industrially important Cu/ZnO:Al (CZA) catalyst is known as a dynamic system adapting to reaction conditions, which renders the application of in situ and *operando* methods key to establish structure function correlations. Herein, a CZA catalyst close to the industrially used compostion was studied using noninvasive and bulk‐sensitive in situ*/operando* microwave cavity perturbation technique and electron paramagnetic resonance spectroscopy during activation and reverse water gas shift reaction. The transient changes of catalytic activity track with the transients of the dielectric properties providing evidence for the importance of bulk properties for catalytic activity. Furthermore, convincing support for the redox reaction mechanism is obtained, and it is shown that H_2_ and CO_2_ uptake is not competing kinetically with each other. In addition, the reservoir of H_2_ and CO_2_ transiently present in the catalyst during catalysis is determined by the chemical potential of the respective reactant, which is directly coupled to the catalytic activity of the system. The findings fit the model of a Schottky barrier at the Cu/ZnO:Al interface, altered by the gas phase composition which in turn alters the catalytic properties of the system.

## Graphical abstract

We investigated the transformations of an industrially relevant CuO/ZnO/Al_2_O_3_ pre‐catalyst during its activation and under operation in reverse water–gas shift reaction. Changes of the dielectric properties induced by H_2_, which acts as an electron donor and oxygen provided by CO_2_ acting as an electron sink are correlated with the catalytic yield, which provides evidence for the importance of bulk properties for catalytic activity of this system.

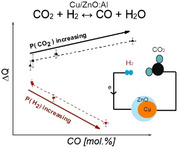

## Introduction

The endothermic reduction of CO_2_ to CO by hydrogen known as the reverse water–gas shift (r‐WGS) reaction has obtained renewed significance in the context of carbon capture and utilization (CCU), particularly for generation of valuable C1 building blocks from anthropogenic CO_2_ emission.^[^ ^1^ ^]^ Ternary Cu/ZnO:Al (CZA) catalysts, which are used industrially as a cost efficient system for large scale synthesis of methanol,^[^ ^2^ ^]^ exhibit good catalytic activity toward CO_2_ reduction and selectivity for CO formation in r‐WGS reaction at ambient pressures.^[^ ^3^ ^]^

Despite extensive investigations on CZA catalysts,^[^ ^2^, ^4^, ^5^, ^6^, ^7^, ^8^ ^]^ aspects such as the reaction mechanism of the r‐WGS reaction,^[^ ^9^, ^10^, ^11^ ^]^ and synergy effects between Cu and ZnO^[^ ^6^ ^]^ remain subjects of ongoing debates. While both Cu and ZnO (with or without Al‐doping) are active in the r‐WGS reaction, CZA is more active than the individual components.^[^ ^12^, ^13^, ^14^ ^]^ The primary suggested reaction mechanisms are redox and associative mechanisms, distinguished by the role of H_2_. In the redox mechanism, CO_2_ is reduced by the activated catalyst and H_2_ serves as a reducing agent to reestablish the active catalyst, while in the associative mechanism H_2_ is involved in CO_2_ dissociation.^[^ ^15^ ^]^ The synergy effect between Cu and ZnO is ascribed to multiple phenomena including wetting/non‐wetting processes of the Cu particles by ZnO in reducing and oxidizing atmosphere, respectively.^[^ ^16^, ^17^, ^18^ ^]^ Furthermore, spillover of hydrogen and oxygen between Cu and ZnO phases is considered important and contribute to the so‐called strong metal support interaction (SMSI) between Cu and ZnO.^[^ ^19^, ^20^, ^21^, ^22^, ^23^ ^]^ In addition, Cu‐ZnO interfaces are suggested to play a central role in electron transfer from ZnO to Cu as they function like a Schottky junction.^[^ ^24^ ^]^

Due to the considerable dynamics of the system, understanding of the abovementioned aspects requires the investigation of the catalyst under operating conditions. In situ/operando measurements provided detailed insight into the large changes in geometric and electronic structure upon activation of the catalyst and also evidence the distinct role of the experimental conditions (gas composition, pressure, and temperature).^[^ ^10^, ^25^, ^26^, ^27^, ^28^, ^29^, ^30^, ^31^, ^32^, ^33^, ^34^ ^]^ While restructuring of an active catalyst can also be observed when reaction conditions are varied, the effects are often rather subtle, even though the catalytic activity changes significantly.^[^ ^28^, ^34^ ^]^ A detailed interpretation of the observed changes is thus still a challenge. Therefore, it is important to explore the potential of alternative physical properties that may reflect the underlying processes more sensitively, specifically to address the developments upon alteration of reaction conditions. To this end, the dielectric properties of catalysts, although being a bulk property, were shown to show changes in response to the exposed gas, measured in a contact‐free manner using the microwave cavity perturbation technique (MCPT).^[^ ^33^, ^35^ ^]^ Importantly, these developments were found to correlate well with the catalytic properties of the system indicating that the latter oftentimes cannot be understood by considering the surface chemistry alone.^[^ ^32^ ^]^ However, the characterization of systems with high dielectric losses, as expected for the CZA catalyst with a Cu loading comparable to that of the industrial catalyst, poses technical challenges, which have prevented application of the method for these systems up to now. The difficulties arise in particular from the necessity to study the catalyst in its various states, i.e., from the calcined pre‐catalyst through activation and under reaction conditions, whereby the dielectric properties change considerably.

In this contribution, an industrial relevant CZA pre‐catalyst containing 68 wt.% CuO (FHI‐standard ^[^ ^36^ ^]^) has been studied via in situ*/operando* MCPT measurements and in situ electron paramagnetic resonance (EPR) spectroscopy. First, the transformation of the CuO/ZnO/Al_2_O_3_ pre‐catalyst (CZA‐prec) to an active Cu/ZnO/Al_2_O_3_ catalyst (CZA) in an activation process was explored. Subsequently, the catalytic properties of the activated system with respect to the r‐WGS reaction were investigated and correlated to the changes of the dielectric properties. Further insight into the underlying processes were obtained from in situ*/operando* MCPT results in H_2_/N_2_ and CO_2_/N_2_ streams.

## Results and Discussion

### Activation of the CZA‐prec

The catalytic activity of the CZA catalyst depends crucially on a variety of parameters among which the activation process of the pre‐catalyst is an important step.^[^ ^26^, ^37^ ^]^ The CZA‐prec material investigated here was prepared by co‐precipitation of a zincian malachite precursor, (Cu,Zn)_2_(OH)_2_CO_3_, followed by calcination of the precipitate at 330 °C, resulting in an amorphous mixed oxide (CZA‐prec) with a total molar metal composition of 68:28:4 (Cu:Zn:Al) containing the oxides CuO, ZnO, and Al_2_O_3_ in a weight ratio of 68:29:3 wt.%. The activation was performed in reducing gas stream of 5 vol.% H_2_ in N_2_ upon heating to 250 °C with a heating rate of 1.2 K min^−1^. More experimental details can be found in the Supporting Information (SI), Experimental Section, and Figures S1–S3.

**Figure 1:**
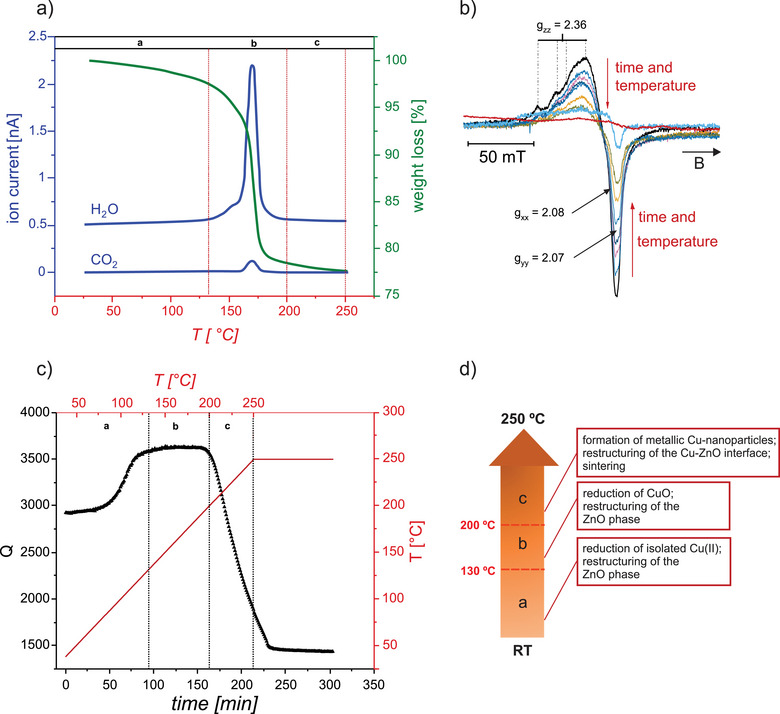
Characterization of the activation process of CZA‐prec under reducing conditions: a) TGA/MS result taken in 5 vol.% H_2_ in He with a heating rate of 1.2 °C min^−1^. b) In situ EPR spectra during the temperature increase (2.4 °C min^−1^) in 5 vol.% of H_2_ in N_2_, black trace: 2 °C, light blue trace: 130 °C, red trace: 150 °C. c) In situ MCPT result in 5 vol.% H_2_ in N_2_ using a heating rate of 1.2 °C min^−1^ up to 250 °C and a holding time of 90 min at 250 °C. Temperatures given at the top aim at an easier comparison with the TGA/MS results in a). Sections a, b, and c correspond to those shown in (a). d) schematics of the main processes occurring during activation of CZA‐prec.

Figure 1a shows the TGA/MS results, obtained during the activation of a fresh CZA‐prec. The mass of the sample decreases from the very beginning of the annealing. While the mass loss is rather moderate (about 3%) up to 130 °C, the curve becomes considerably steeper with a maximum loss rate at about 175 °C. Above 180 °C, the curve flattens and about 90% of the weight loss has taken place at 200 °C. The large weight loss around 175 °C is accompanied by a large amount of water desorption and a visible but much smaller desorption of CO_2_. Water formation is expected due to the reduction of Cu(II)O. In addition, desorption of CO_2_ has previously been assigned to the decomposition of residual hydroxy carbonate species ((Cu,Zn)_2_(OH)_2_CO_3_) or so‐called high temperature carbonate phases.^[^ ^34^, ^36^ ^]^

A closer look at the desorption trace of water in the mass spectrometer shows that only small amounts of water are desorbed below about 130 °C. The amount of water desorption increases significantly above 140 °C forming a shoulder to the main desorption peak at about 170 °C. Based on previous studies of the activation of calcined CZA pre‐catalysts containing Cu(II)‐oxide as well as varying proportions of ZnO and carbonate containing phases, it is concluded that the system undergoes reductive formation of metallic Cu particles and a restructuring of the Zn‐containing phases within this temperature range.^[^ ^28^, ^34^ ^]^ The process depends on the details of the procedure such as temperature profile or gas composition. It was for example shown that an increase in H_2_ pressure reduces the temperature at which reduction of CuO occurs.^[^ ^28^ ^]^

To elucidate the reduction of the Cu(II)‐species in more detail, in situ EPR spectra were taken during the activation process in the temperature range between 25 and 150 °C, as shown in Figure 1b. The room temperature EPR spectrum of the pristine CZA‐prec (black trace) shows the highest intensity within the series, and the line shape is compatible with the expectation for a system containing Cu(II) ions.^[^ ^38^, ^39^, ^40^ ^]^ In this respect, the maxima observed at low field are characteristic for the hyperfine interaction of the electron spin (g_z_‐component g ≈ 2.36, *S* = ½) with the nuclear spin of both naturally abundant isotopes of copper (^63^Cu and ^65^Cu, *I* = 3/2). The spectra clearly show the two low field signals, while the other two signals of the hyperfine manifold cannot be discriminated from the broad signal. The two other g‐tensor components form an almost axial g‐tensor (*g* = 2.07–2.08), which results in the sharp high field minimum of the spectrum. However, a more detailed analysis of the line shape reveals that the observed spectrum cannot be understood assuming an ensemble of isolated Cu(II) ions (see Figure S4 and explanations in Chapter 1 of the Supporting Information), which is in‐line with expectations for a calcined CZA‐prec, containing small CuO nanoparticles. CuO is paramagnetic at room temperature and its EPR spectrum depends crucially on the structural and electronic properties of the material. The spectrum is typically characterized by broad lines, which are explained by exchange coupling between the paramagnetic ions in the lattice.

The intensity of the EPR signal decreases monotonously (see Figure S5) with increasing temperature and the highest temperature at which a signal similar to that of Cu(II) ions occurs is at about 130 °C (light blue trace). The subsequent spectrum taken at about 150 °C lacks clear evidence for the presence of isolated Cu(II) ions. Importantly, this is below the temperature at which significant water desorption associated with the main reduction of the pre‐catalyst is observed in the TGA/MS. However, a mass loss of a few percent is observed in this temperature range, which is consistent with a partial reduction of the system. As the observed EPR spectrum accounts only for the fraction of magnetically isolated Cu(II) species, the experimental results are consistent with a reduction of these Cu(II) species to diamagnetic Cu(I) ions or metallic Cu. However, a restructuring of the sample leading to a magnetic coupling of the Cu(II) ions, e.g., by segregation of Cu(II) ions out of the ZnO lattice into the CuO phase cannot be excluded. It is, however, clear that the speciation of the Cu ions is altered at temperatures well below the main reduction temperature, which evidences significant dynamics within the system.

Apart from the EPR signals assigned to Cu(II) species, the sample lacks other EPR active species. In particular, ZnO defects observed for Cu/ZnO systems at g‐values around 1.960, typically attributed to shallow donor sites in the ZnO crystal lattice,^[^ ^33^ ^]^ are not observed. This is consistent with the reported decrease in the amount of ZnO lattice defects with increasing Cu‐content,^[^ ^38^ ^]^ suggesting that higher Cu fraction in the pre‐catalysts may lead to a greater incorporation of Cu‐ions in the ZnO:Al structure.^[^ ^38^, ^41^ ^]^

Figure 1c shows the result of the in situ MCPT experiment in which the CZA‐prec was heated in 5 vol.% H_2_ in N_2_ using the heating rate of the TGA experiment, and subsequently held at 250 °C for another 90 min. The Q‐value, which is a measure for the dielectric losses of the system (for the definition and calculation of the Q‐value, see Supporting Information), increases with increasing temperature in the range between room temperature and about 130 °C. Upon further increase of the temperature, the Q‐value is almost constant (∼3600) up to about 200 °C, above which the Q‐value drops drastically. Please note that the steep decrease of the Q‐value ends only after the sample was at the maximal temperature of 250 °C for about 30 min indicating that the associated processes are rather slow at this temperature.

The results of the MCPT measurement can be correlated with the results of the TGA/MS experiment (see temperature sections a, b, and c in Figure 1a and c). According to TGA, heating the sample to around 130 °C results in a weight loses of a few percent, which is largely due to water desorption (Figure 1a). The decrease in dielectric losses observed in this temperature range by MCPT (Figure 1c) is consistent with a loss of physisorbed water, which is known to absorb microwaves very strongly through a mechanism known as dipole polarization.^[^ ^42^ ^]^ However, the EPR results show that desorption of adsorbed water alone is not sufficient to explain the increase of the Q‐value associated with a decrease in dielectric losses as the speciation of Cu ions is also changing in this temperature range (section a in Figure 1a and c). The temperature interval between room temperature and 130 °C is below the onset of the main reduction of the system, which is associated with the transformation of CuO into metallic Cu‐nanoparticles.^[^ ^28^, ^34^ ^]^ The comparatively low water release in this temperature range (Figure 1a) in combination with the change in the EPR signal (Figure 1b), and the strong change in the Q value (Figure 1c) suggests that structural rearrangements in the Zn‐containing phases occur already below the main reduction of the CuO particles. To what extend these changes in the nanostructured material contribute to the reduction in dielectric losses is something that can currently only be speculated about.

In TGA/MS, the main reduction is observed in section b, in particular between 160 and 180 °C (Figure 1a), which is well in‐line with previous measurements using complementary techniques such as in situ DRIFT spectroscopy.^[^ ^27^ ^]^ Interestingly, the Q‐value remains almost constant in this range. This behavior is not expected as Cu metal nanoparticles with sizes well below the skin depth of copper (about 3 µm) should result in dielectric losses of the sample, induced by the excitation of conduction band electrons.^[^ ^43^ ^]^ The structure of the Cu nanoparticles is found to be complex. The average particle size of Cu in CZA catalysts was determined to be around 7–9 nm by TEM, and the size of crystalline Cu domains obtained from neutron diffraction patterns was about half of that size.^[^ ^36^ ^]^ The constant Q value in the range between 130 and 200 °C suggests that metallic copper is initially formed in quasi‐atomic dispersion or in the form of very small nanoparticles in a defect‐rich state. The subsequent decrease in Q in the temperature range between 200 and 250 °C and under isothermal conditions at 250 °C is tentatively attributed to sintering into metallic Cu nanoparticles and healing of defects.

The structural changes in the system are not restricted to the formation of metallic Cu‐nanoparticles. Importantly, the Cu particles form a specific interface with the ZnO‐phases and were also shown to be encapsulated by a thin amorphous ZnO layer after the reductive activation of the CZA‐prec.^[^ ^26^, ^27^, ^44^ ^]^ Apart from the losses due to excitation of conduction band electrons in the Cu nanoparticles mentioned above, the heterojunction, created between the Cu metal and the ZnO semiconductor acts like a Schottky barrier.^[^ ^24^ ^]^ The redistribution of charge carriers at such heterogeneous interfaces induce Maxwell–Wagner polarization whose interaction with the altering electromagnetic (EM) field is also known to contribute to the dielectric losses.^[^ ^45^ ^]^ To this end, changes in the electronic structure of ZnO, e.g., through dissolution of hydrogen, will affect not only the conductivity of ZnO but also the charge carrier distribution at the heterojunction, and thus the contribution of the Maxwell–Wagner polarization to the dielectric losses. Therefore, changes in the nano‐ and defect structure of ZnO can also contribute to the drop of Q at higher reduction temperatures. Figure 1d summarizes the main developments observed during the transformation of CZA‐pre to the CZA catalyst during the activation process in hydrogen.

### MCPT During R‐WGS Reaction

**Figure 2:**
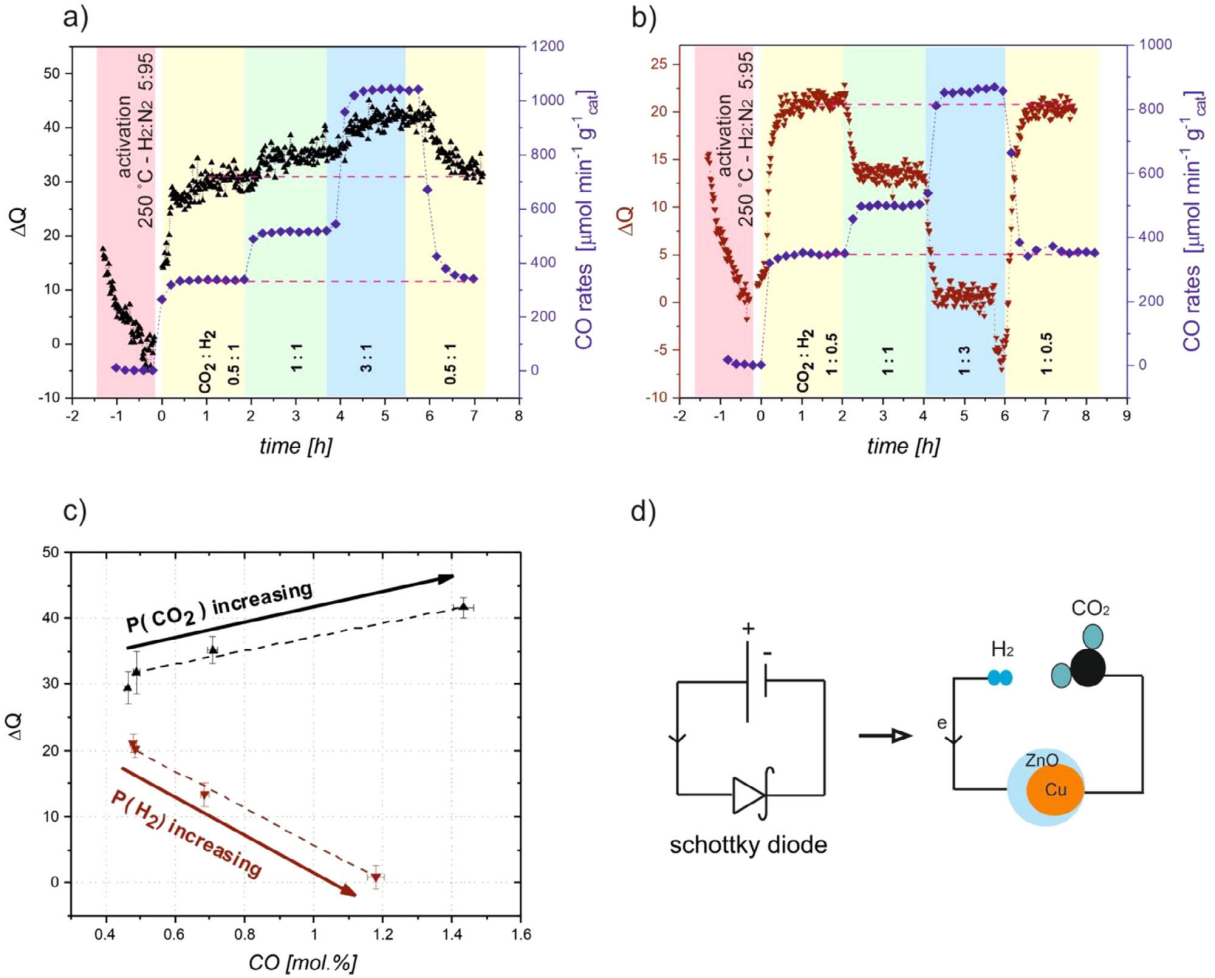
*Operando* MCPT measurement of CZA catalyst in gas mixtures of r‐WGS reaction (CO_2_/H_2_/N_2_) at 230 °C: a) increasing CO_2_ partial pressure; b) increasing H_2_ partial pressure; c) correlation between steady state ΔQ and CO yield upon variation of CO_2_ (black symbols) and H_2_ (red symbols) partial pressure, (data from (a) and (b)). d) schematic electrical circuit of the reaction. H_2_ and CO_2_ as reducing and oxidizing agents, respectively, along with the CZA catalyst as a Schottky diode.

*Operando* MCPT experiments probing the catalytic performance of the activated CZA catalysts with respect to the r‐WGS reaction in a CO_2_/H_2_ containing gas mixture at 230 °C are depicted in Figure 2a,b. In these experiments, the partial pressure of one of the reactants (H_2_ in a and CO_2_ in b) is fixed at 100 mbar (10 vol%), while the partial pressure of the other reactant is varied, leading to two series of experiments with CO_2_:H_2_ ratios of 1:0.5, 1:1, 1:3, and back to 1:0.5. Analysis of the GC measurements shows that the selectivity of CO is close to 100% with traces of methanol as the only side product. The CO yield (and selectivity) is very comparable to the one previously reported for a similarly prepared catalyst.^[^ ^36^ ^]^ For a constant H_2_ partial pressure, an increase in CO production is observed with increasing CO_2_ pressure (Figure 2a), which is also observed when the H_2_ partial pressure is increased at constant CO_2_ partial pressure (Figure 2b). A steady state reactivity with no indication for deactivation is established for each gas composition and switching to the initial one at the end of the series leads to the same catalytic activity indicating that the changes resulting in the catalytic properties are reversible. From the steady state rates, positive apparent reaction orders of 0.5 and 0.59 were determined with respect to H_2_ and CO_2_, respectively (see Figure S6). Apparent reaction orders for H_2_ and CO_2_ smaller than one and a higher reaction order toward CO_2_ are in agreement with previous kinetic studies of similar Cu‐based catalysts in the r‐WGS reaction,^[^ ^33^ ^]^ proving that the reactor design employed in the work allows the determination of catalytically relevant parameters.

The catalytic activity is mirrored by the results of *operando* MCPT measurements. Increasing the CO_2_, partial pressure (Figure 2a) leads to an increase of the Q‐value and a reversible reduction of it after switching back to the initial gas composition. Changes of the Q‐value upon increasing H_2_ partial pressure are also perfectly reversible after returning to the initial gas composition. However, an increase in H_2_ partial pressure leads to a decrease of the measured Q‐values, which is about twice as large as the increase observed upon increasing the partial pressure of CO_2_.

The transients of the Q‐value track nicely with the temporal evolution of the CO‐yield. By applying a simple exponential kinetics model to the transients, time constants for the process were determined, which were ranging between 7–10 min (see Figure S7, the fittings correspond to the switch to a CO_2_:H_2_ ratio of 1:1 shown in Figure 2b, as an example). Furthermore, Figure 2c shows that Δ*Q*, defined as Q_(reaction)_‐Q_(activation)_ (see also Supporting Information for more explanation of Δ*Q*) in the steady state, correlates linearly with the yield of CO. These results clearly show that the catalytic activity is directly linked to the processes, leading to the changes in the dielectric properties. The inverse trends in Figure 2c are consistent with the concept that H_2_ acts as a reducing agent, i.e., as an electron donor, whereas CO_2_ acts as an oxidizing agent, i.e., as an electron acceptor. In this context, the metal‐semiconductor interface between Cu and ZnO:Al in CZA catalysts, which acts as a Schottky barrier,^[^ ^24^ ^]^ can be altered by injection or depletion of charge carriers. A schematic of the corresponding model is depicted in Figure 2d. In order to gain additional insight into the microscopic reasons and underlying processes resulting in the observed changes in the dielectric properties, the MCPT results obtained for the activated CZA catalysts exposed to the individual reactant gases are discussed below.

### MCPT in H_2_/N_2_ and CO_2_/N_2_ Streams

**Figure 3:**
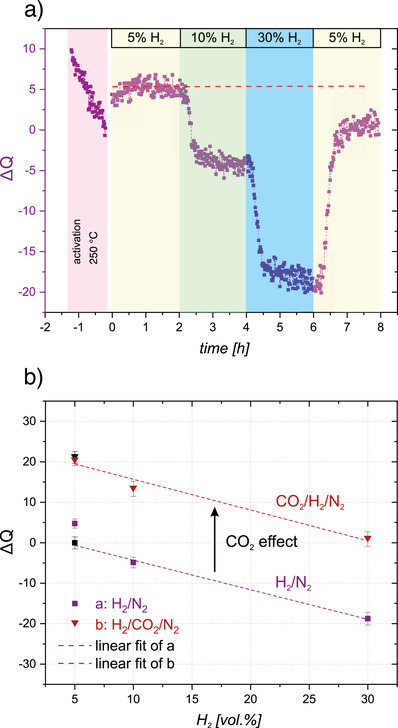
a) In situ MCPT measurement of Δ*Q* in a H_2_/N_2_ stream at 230 °C with increasing partial pressure of H_2_ and b) comparison of Δ*Q* changes with varying partial pressure of H_2_ in the H_2_/N_2_ and H_2_/CO_2_/N_2_ streams at 230 °C. The black data points are related to the reversibility checks in the last section of the experiments.

In situ MCPT measurements of the activated CZA catalyst at 230 °C as a function of H_2_ partial pressure in a N_2_ stream are depicted in Figure 3a. Switching the temperature from 250 °C (final activation temperature) to 230 °C in 5 vol.% H_2_ in N_2_ leads to an increase in the Δ*Q*‐value of about 4. A stepwise increase of the hydrogen partial pressure from 5 vol.% to 10 vol.% and 30 vol.% results in a stepwise decrease of Δ*Q*, which is found to stabilize rather shortly after switching to the different gas feeds. Going back to the initial low partial pressure of H_2_ in the final step of the experiment shows that the changes induced by H_2_ are largely reversible (see red dashed line in Figure 3a). Hence, the system rather quickly establishes an equilibrium with the gas phase. Please note that the transient kinetics of the underlying processes are comparable to the ones observed upon the same changes of H_2_ partical pressure during r‐WGS reaction in the series shown in Figure 2b. The reversibility of the changes excludes a significant loss of oxygen as expected if an increased hydrogen pressure would result in additional vacancy formation in ZnO as water expected to be formed would desorb and would thus lead to irreversible changes in the absence of an oxygen source. The decrease of the Q‐values with increasing H_2_ partial pressure is consistent with the observations during r‐WGS reaction and previous findings on Cu‐ZnO based catalysts and ZnO.^[^ ^33^, ^46^ ^]^

As the oxygen content of the system stays constant, the decreasing Q‐values are in‐line with expectations based on direct current (DC) conductivity and contact‐free MW conductivity measurements showing increasing conductivity of ZnO upon hydrogen uptake,^[^ ^33^, ^47^ ^]^ which was associated with the formation of shallow donor states based on DFT calculations.^[^ ^48^ ^]^

The change of Δ*Q* correlates linearly with the hydrogen partial pressure (Figure 3b, purple squares). The same trend is observed for the H_2_ pressure dependence during the r‐WGS reaction (Figures 2b,c and 3b, red triangles). Please note that the change in the Q‐value is not only linearly dependent on the H_2_ partial pressure as under r‐WGS conditions but both curves have also the same slope. For the given CO_2_ partial pressure of 100 mbar, a constant shift of Δ*Q* of about 20 is found.

**Figure 4:**
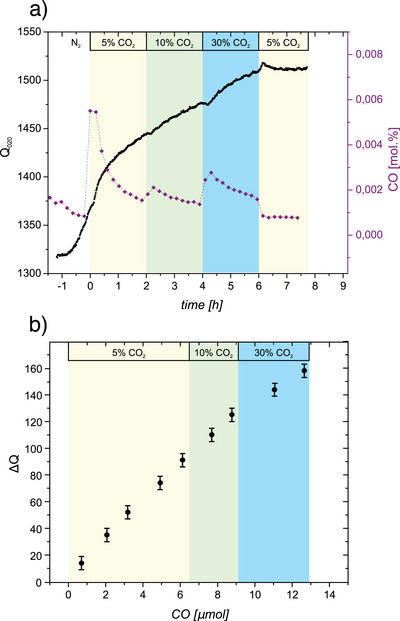
a) *Operando* measurement of Q‐values (Q_020_) and yields of CO in CO_2_/N_2_ stream by increasing partial pressure of CO_2_ at 230 °C. b) Correlation between the change of the Q‐value (△Q) and the amount of produced CO as deduced from the data in (a).

To investigate the effect of CO_2_ on dielectric loss properties of the CZA catalyst, an *operando* MCPT measurement in CO_2_/N_2_ mixtures with increasing partial pressure of CO_2_ (5, 10, 30, and back to 5 vol.%) was conducted at 230 °C (Figure 4a). After the activation process, the reactor was purged with pure nitrogen. Exposing the activated CZA catalyst to CO_2_ containing atmosphere (5 vol.% CO_2_ in N_2_) results in a gradual increase of the Q‐value from about 1350 to 1450 (Δ*Q* ≈  100), which is a significantly larger increase as compared to the changes in Δ*Q* observed during r‐WGS reaction conditions with the same partial pressure of CO_2_ (Figure 2a, yellow parts Δ*Q* ≈  30). Additionally, no stable Q‐value is established during 2 h on stream, whereas a stable Q‐value was established during r‐WGS reaction within about 30 min. By increasing the CO_2_ partial pressure to 10 vol.% and subsequently 30 vol.%, the Q‐values increase further but are never stabilized during the 2 h exposure of the catalyst to each gas mixture. Only after reducing the partial pressure of CO_2_ back to the initial value of 5 vol% the Q‐value stabilizes to the final value at the end of the period with 30 vol% CO_2_ in the gas mixture. This behavior is in sharp contrast to the one observed under r‐WGS reaction as the CO_2_ induced changes are not only significantly larger but also entirely irreversible. This excludes the contribution of adsorbates such as CO_2_^ᵟ−^, which were considered to alter the charge distribution at the heterojunction and thus the dielectric properties, as a change in gas phase composition should alter the coverage of these species.^[^ ^32^ ^]^ The reason for irreversibility becomes clear in combination with the GC results showing that the increase in Q‐values is associated with a transient CO production, i.e., a reaction of CO_2_ with the catalyst. An analogous behavior of CO production in 5% CO_2_ in N_2_ using a Cu‐ZnO based catalyst was reported and interpreted as evidence for the redox reaction mechanism in the r‐WGS reaction, i.e., the reduction of CO_2_ to CO on the CZA catalyst, followed by the formation of water from the oxygen transiently stored in the catalyst.^[^ ^49^ ^]^ The data provided in Figure 4a allow to correlate Δ*Q* with the amount of CO produced (Figure 4b). There is an almost linear correlation between the amount of CO produced and Δ*Q*. With respect to a correlation between CO yield and Q‐value, it is important to note that the CO production drops to zero (see CO level at the beginning and the end of the colored regions in Figure 4a) after switching to 5 vol% CO_2_ at the end of the experiment. In combination with the largely reversible behavior of the Q‐values due to H_2_/N_2_ streams (see Figure 3a), which excludes a loss of oxygen, the linear correlation between the change of the Q‐value and CO production allows to correlate the changes of the Q‐value with changes in the oxygen content during catalysis. Thus, it allows to quantify the amount of oxygen transiently stored in the activated, i.e., reduced catalyst as a function of reaction conditions.

## Discussion

The MCPT results observed during the r‐WGS reaction shows that the dielectric properties of the system adopt a steady state which depends on the different feed compositions, i.e., ratios of CO_2_ and H_2_ partial pressure. The changes during r‐WGS reaction were found to be reversible, which also holds for changes of the dielectric properties induced by H_2_/N_2_ mixtures. On the contrary, no steady state is reached (on the time scale of the experiment) in case the activated (reduced in H_2_/N_2_ feed) catalyst is exposed to a CO_2_/N_2_ atmosphere as the corresponding changes of the dielectric properties in CO_2_/N_2_ are irreversible. The associated changes in the dielectric properties scale linearly with the transient production of CO, which allows to conclude that the change in dielectric loss is due to an uptake of oxygen atoms as CO_2_ is reduced to CO and allows to quantify their amount. This has important implications for the result presented in Figure 3b, which shows a constant increase of the Q‐value of about 20 with different partial pressures of H_2_ in N_2_ when compared to the corresponding dependence during r‐WGS reaction (variation of the H_2_ pressure at a constant CO_2_ pressure of 100 mbar). According to Figure 4b, this change in the Q‐value (ΔQ) corresponds to the formation of about 1 µmol of CO, which relates to less than 0.5 mol% of oxygen atoms transiently taken up by the catalyst upon addition of 100 mbar CO_2_ to the gas mixture. *Operando* X‐ray spectroscopic methods providing element specific information have been used to study the changes of the CZA catalyst between the activated state and a catalytically active state for r‐WGS reaction.^[^ ^28^, ^34^ ^]^ Both states were found to exhibit very similar spectroscopic signatures with small changes observed for both Zn and Cu near edge features. While a detailed interpretation of these small changes was not possible, they are perfectly consistent with the small amount of oxygen transiently taken up by the system in the catalytically active state, which is observed in the present experiments. Importantly, the MCPT result presented here clearly demonstrates the high sensitivity of the *operando* MCPT method to monitor and quantify the redox processes in the system. In light of the small changes in oxygen content between the different gas feed compositions, the small changes, which were observed in previous X‐ray‐based *operando* studies, can be understood.^[^ ^50^, ^51^, ^52^ ^]^

Along the same lines, the constant shift of the Q‐values for a fixed CO_2_ pressure and varying H_2_ pressures (Figure 3b) evidence a constant amount of transient oxygen species, independent of H_2_ partial pressure, implying that the amount of oxygen uptake is determined by the partial pressure of CO_2_. Conversely, the identical decrease in Q with increasing H_2_ partial pressure also implies that the hydrogen induced increase in dielectric losses is determined by the chemical potential of hydrogen and is not altered by the presence of CO_2_ since the hydrogen induced changes in Q are reversible (no loss of oxygen content). If the transient oxygen concentration is determined by the CO_2_ partial pressure, an increased amount of oxygen is expected with increasing CO_2_ pressure (at constant H_2_ pressure). This is in perfect agreement with the experimental results presented in Figure 2a, i.e., an increase in the Q‐value with increasing CO_2_ partial pressure. Now, if the amount of oxygen and hydrogen present in the catalyst under catalytic turnover conditions is controlled independently by the partial pressures of CO_2_ and H_2_, respectively, the corresponding processes responsible for establishment of the steady state reservoirs cannot compete kinetically with each other. In case surface coverage is not small, this would imply that the two processes take place at different sites. While these results do not allow to draw more detailed conclusions about the location of the corresponding sites, it has been possible to show using in situ*/operando* EPR spectroscopy that ZnO:Al in the absence of metallic Cu reacts with both H_2_ and CO_2_. However, the amount of oxygen, which can be stored in a reduced ZnO:Al sample by reaction with CO_2_, is considerably smaller than for the CZA catalyst, suggesting either a direct contribution of the Cu particles to CO_2_ reduction or the role of Cu particles in promoting ZnO as a catalyst for r‐WGS reaction.^[^ ^14^ ^]^

Increasing the CO_2_ partial pressure from 50 to 300 mbar (Figure 2a) corresponds to an increase in Δ*Q* of about 10, which is associated with an increase in the oxygen content in the low permille‐range. At the same time, CO formation increased by a factor of about three. As shown above, the amount of hydrogen transiently present in the system is determined by the partial pressure of hydrogen in the gas phase. Thus, the same number of hydrogen species in the system results in an increased CO_2_ reduction with increasing CO_2_ pressure. Conversely, an increased CO_2_ reduction is also observed for an increasing partial pressure of H_2_ at a fixed partial pressure of CO_2_. Please note that the amount of transiently stored oxygen atoms remains constant under these conditions (Figure 3b).

These aspects allow to draw some additional conclusions. In case, the CO_2_ partial pressure was kept constant, an increasing steady state formation of CO is observed with increasing H_2_ pressure, i.e., with an increasing amount of hydrogen species in the system. While it is not surprising that an increasing amount of hydrogen allows for an increasing reaction rate, it is important to keep in mind that the increasing reaction rate does not reduce the amount of transient oxygen species. As the latter reservoir is coupled to the catalytic activity, the reduction step of CO_2_, which supplies oxygen atoms to fill this reservoir, must take place so quickly that a change in the reaction rate by a factor of 2.5 does not alter its steady state concentration. On the other hand, for a given amount of hydrogen in the system, as set by its partial pressure in the gas phase, increasing the CO_2_ pressure also leads to an increase in the steady state reaction rate, which shows that sufficient hydrogen is available to establish the new steady state. However, under these conditions the transient concentration of oxygen species increases with increasing reaction rate. Within the redox mechanism, a possible explanation to reconcile these observations would be an increased number of reactive surface oxygen species. These additional species are formed only after increasing the chemical potential of CO_2_ in the gas phase. This suggests that additional surface sites become available for CO_2_ reduction which require the increased chemical potential, i.e., are less favorable for this reaction. In line, with the Evans–Bell–Polanyi principle, the presence of such surface oxygen species may facilitate water formation as long as hydrogen is available. It is important to note that a positive reaction order is observed for both CO_2_ and hydrogen, which shows that the availability of hydrogen is not strongly limiting the reaction rate. In turn, this shows that hydrogen transport, which includes hydrogen spill over between the Cu and ZnO:Al phases, suggested to take place in this system,^[^ ^19^, ^20^, ^21^ ^]^ is still sufficiently fast to establish an increased steady state reaction rate with increasing CO_2_ pressure.

With respect to the microscopic origin of the catalytic activity of the system, it is interesting that the transient evolution of the reaction rate, which is paralleled by the change of the dielectric properties, approaches a new steady state after switching conditions on the time scale of about 10 min, which is slow as compared to the rate at which molecules are impinging on the surface, indicating that some slow processes are involved. Please note that comparable time constants are observed for changes of the dielectric constants when changing the partial pressure of H_2_ in H_2_/N_2_‐feeds (Figure 3a) as well as for a reduction of the H_2_ partial pressure from 30% to 5% (Figure 2b). H_2_ TPD of CZA catalysts^[^ ^53^, ^54^ ^]^ shows H_2_ desorption below 180 °C. Consequently, the surface coverage of hydrogen will adapt rapidly to changes in the partial pressure of hydrogen in the gas phase. As the transient catalytic activity exhibits a time constant of around 10 min after reducing the hydrogen partial pressure from 30% to 5%, the transient change in catalytic activity cannot be explained by changes in surface hydrogen coverage. However, it does track the transient changes in dielectric properties. A possible explanation could be diffusion of hydrogen into and out of the bulk of ZnO, which was found to be a rather slow process.^[^ ^55^, ^56^ ^]^ Employing environmental TEM, Hansen et al. have shown that changes in the gas composition can lead to reversible structural changes as observed for isothermal switching from a H_2_ to H_2_/H_2_O mixtures and back to H_2_‐containing gas feed.^[^ ^25^ ^]^ Even though reversible structural changes were only shown for systems with basically unperturbed Cu/ZnO interface, the dynamic changes in particle shape were already previously considered an important aspect to understand the catalytic properties of the CZA system.^[^ ^57^ ^]^ Changes in the geometric structure are certainly important for a detailed understanding of the catalytic activity, but small reversible geometric changes in the Cu particle shape observed by environmental TEM cannot readily explain the observed changes in the dielectric losses, which perfectly match the transient of the catalytic activity. In addition, the steady state r‐WGS activity of CZA studied here is linearly correlated with the changes in dielectric losses. Given that the dielectic losses are known to depend on the properties of the the Schottky barrier present at the Cu/ZnO interface, and given the impact of this barrier on important reaction steps such as the activation of CO_2_,^[^ ^32^ ^]^ it is reasonable to conclude that the observed changes in the dielectric properties play a crucial role for the catalytic activity, rather than merely reporting on effects associated with geometric changes of the system. The correlation of electronic modifications of the Cu/ZnO interface with the catalytic properties of the system may also have implications for other reactions such as methanol synthesis.^[^ ^2^, ^17^, ^25^ ^]^ With respect to an understanding of the catalytic properties, this effect acts as yet another aspect (in addition to, e.g., structural rearrangements), which would have to be taken into account, e.g., by dynamic microkinetic modeling.^[^ ^57^, ^58^ ^]^ While *operando* MCPT provides experimental evidence for the transient incorporation of oxygen into the system and thus for the presence of the central step in the redox mechanism, it is important to keep in mind that the evidence for the involvement of the redox mechanism does not allow to exclude other reaction pathways.

## Conclusion

*Operando/*in situ MCPT and EPR spectroscopic measurements were employed to investigate a CZA catalyst with composition close to that of the industrially used system. A combination of these two techniques with TGA/MS measurements provide evidence for a rather complex behavior of the calcined material during activation in hydrogen. While EPR spectroscopy provides clear evidence for a change in the Cu(II) speciation at temperatures well below the main reduction temperature, the dielectric properties probed by in situ MCPT show a distinct decoupling of the chemical reduction of the system, taking place at around 170 °C as deduced from the TGA/MS, from the evolution of electronic properties of the catalyst, which were shown to require at least 30 min at 250 °C to come close to a stable state. On the one hand, it illustrates the high sensitivity of MCPT to probe changes in such complex material systems, and on the other hand, it provides insights as to why complex activation protocols are required to arrive at a high‐performance catalyst as chemical transformation is necessary but not sufficient to establish a functional system.

In situ and operando MCPT was further employed to gain insight into the dynamics of the CZA catalyst during r‐WGS reaction. It was found that the changes in the dielectric losses observed as a function of the partial pressures of both reactant gases are linearly correlated with the yield of CO in the r‐WGS reaction. In combination with the correlation of the transient trends, it proves that the bulk properties of the catalyst are key for the catalytic performance. The increasing losses with increasing partial pressure of H_2_ and the reduction of dielectric losses with increasing partial pressure of CO_2_ could be related to the ability of H_2_ to act as a source of charges whereas reaction with CO_2_ was found to oxidize the system, thus acting as a charge sink. Based on the results obtained from exposing the CZA catalyst to the reactants (H_2_/CO_2_) individually, it was possible to show that the observed changes of the Q‐value during r‐WGS reaction can be associated with a transient uptake of oxygen by the catalyst. This result not only provides clear evidence for the redox mechanism, as oxygen atoms are deposited into the system, but it also allows to quantify the amount of oxygen atoms the activated catalyst stores transiently in the catalytically active state. For the conditions investigated, here, the amount is well below 1 mol% of the total oxygen content in the system. It is also important to note that the reservoirs of hydrogen and oxygen present during catalytic activity, which determine the dielectric properties, are not only independently defined by the partial pressures of the reactant gases but are also correlated to the catalytic activity of the system. The analysis of these results considering the catalytic properties, i.e., the positive reaction order in both H_2_ and CO_2_, allows to conclude that the sites for hydrogen and oxygen uptake do not kinetically compete with each other. While evidence for contributions of Cu and ZnO:Al phases for the corresponding uptake exists, the results obtained here do not allow for further insight into their relative importance. From the positive reaction order in both H_2_ and CO_2_ in combination with *operando* and in situ MCPT results, it can be concluded that hydrogen transport is not limiting catalytic activity and that the system exhibits sites of different reactivity towards CO_2_ reduction, whose participation during r‐WGS reaction depends on the gas phase composition. The parallel evolution of the transients in catalytic activity and dielectric properties indicates that the latter is a crucial descriptor for the catalytic properties, which aligns with the model that the Schottky barrier at the Cu/ZnO:Al interfaces plays an important role for the catalytic activity.

## Supporting Information

The authors have cited additional references within the Supporting Information.^[^ ^59^, ^60^, ^61^, ^62^, ^63^, ^64^, ^65^, ^66^ ^]^

The data that support the findings of this study are openly available in Zenodo at https://doi.org/10.5281/zenodo.14904233.

## Conflict of Interests

The authors declare no conflict of interest.

## Supporting information



Supporting Information

## Data Availability

The data that support the findings of this study are openly available in [Zenodo] at [https://doi.org/10.5281/zenodo.14904233], reference number [0].
