# Supplementary material for: Revealing the Active State of a Cu/ZnO:Al Catalyst During Reverse Water–Gas Shift Reaction in an Operando Microwave Absorption Study
Source: Angew Chem Int Ed Engl. 2025 Sep 30;64(46):e202504280. doi: 10.1002/anie.202504280 (PMC12603976; doi:10.1002/anie.202504280)
Supplement: Supplementary file 1 — Supporting Information [file ANIE-64-e202504280-s001.pdf]

Supporting Information

Revealing the Active State of a Cu/ZnO:Al Catalyst during Reverse Water-Gas Shift Reaction in an Operando Microwave Absorption Study

Zohreh Asadi[a], Clara Patricia Marshall[b], Annette Trunschke[b], Thomas Risse[a*]

[a] *Institute of Chemistry and Biochemistry, Freie Universität Berlin, Arnimallee 22, 14195 Berlin, Germany*

[b] *Department of Inorganic Chemistry, Fritz-Haber-Institut der Max-Planck-Gesellschaft, Faradayweg 4–6, 14195 Berlin, Germany*

E-mail: *risse@chemie.fu-berlin.de*

Table of Contents

1. Experimental Section 1

Catalyst synthesis and characterization 1

Operando apparatus 3

Quality factor 4

MCPT measurements 5

EPR measurements 6

2. Temporal evolution of Q-value and catalytic activity 10

References 10

1. Experimental Section

Catalyst synthesis and characterization

The CZA-prec (FHI-code: S36204) was synthesized following the protocol outlined in a previous work by Schumann *et al.*[1] In brief, the precursor was synthesized by co-precipitation at 65°C from a mixed aqueous Cu, Zn, Al nitrate solution (1 M metal based), which was acidified by addition of 10 ml of concentrated HNO_3 (65%). For this purpose, 164.29 g $Cu(NO_3)_2\cdot 3\ H_2O$, 86.28 g $Zn(NO_3)_2\cdot 6\ H_2O$ and 11.22 g $Al(NO_3)_3\cdot 9\ H_2O$ were dissolved in 1000 mL HNO_3-contaning deionized water (MilliPore®). The precipitation was conducted in a custom designed automated laboratory reactor (2 L synthesis workstation RX-10, Mettler-Toledo GmbH), using an 1.6 M Na_2CO_3 solution as basic precipitating agent prepared by dissolving 171.32 g of anhydrous Na_2CO_3 in 1000 mL water. The thermostated glass reactor was filled with 400 mL water, and 600.1 g of the mixed metal solution were dosed over 30 min. The simultaneous addition of the precipitating agent (424.1 g) was controlled by the measured pH value so that the pH remained constant at 6.5. A blue suspension was obtained, which was aged in the reactor for 50 min at 65°C until a pH drop was observed and the color of the suspension changed to green. The precipitate was filtered (15A) and washed 4 times with 1 L distilled water until the conductivity was below 0.5 mS/cm (measured value 0.38 mS/cm). Finally, the green solid was suspended in 600 mL water and spray-dried (settings: T_{inlet} = 180°C, T_{outlet} = 80°C, flow rate = 45 $mL\cdot min^{-1}$, pump = 50-60%) in a Büchi Mini Spray Dryer B-290 to give 50.6 g of a green powder (FHI-code: S32774). An amount of 5.02 g of the green powder was calcined at 330 °C (heating rate 2 $K\cdot min^{-1}$) in a quartz tube in a pendulum furnace (Xerion) for 180 min in a mixture of 25 $mL\cdot min^{-1}$ O_2 and 100 $mL\cdot min^{-1}$ Ar to give 3.6 g of the black pre-catalyst powder.

The chemical composition of the pre-catalyst was determined by X-ray fluorescence spectroscopy applying a Brucker S8 TIGER X-ray spectrometer. For sample preparation, a mixture of 100 mg of the catalyst and 8.9 g of lithium tetraborate (>99.995%, Aldrich) was fused into a disk using an automated fusion machine (Vulcan 2 MA, Fluxana). The composition of CuO : ZnO : Al_2O_3 is 68.1 : 29.0 : 2.9 (mass percent).

The specific surface area was determined for CZA-prec (FHI code: S35214), which is a replica of the calcination of the precipitate (FHI code: S32774), using nitrogen adsorption at -196ºC. The measurements were carried out in a Quantachrome Autosorb 6B gas sorption system after outgassing the sample under vacuum for 2 hours at 100°C and measuring full adsorption-desorption isotherms (79 data points). The specific surface area of 109 m²/g was calculated using the Quantachrome Autosorb software package and applying the multipoint Brunauer–Emmett–Teller (BET) method in the range 0.05 < p/p_0 < 0.3 assuming a N_2 cross sectional area of 16.2 $Å^2$. The sample contains mesopores with an average pore diameter of 23 nm and

a pore volume of 0.8 $cm^3 \cdot g^{-1}$ according to the analysis of the desorption branch of the isotherm by the Barrett, Joyner and Halenda (BJH) method.

X-ray diffraction (XRD) patterns were measured in 2 θ from 10–135° with a step size of 0.02° using a Bruker D8 Advance A25 diffractometer operated at 40 kV and 40 mA using Cu-Kα radiation and a position sensitive energy dispersive LYNXEYE XE-T silicon strip detector in high energy resolution mode (no Ni filter required). The freshly calcined sample is poorly crystalline and shows broad reflexes at about 35 and > 50 2 θ for CuO (Figure S1). Other components are ZnO (shoulder between 30 and 35 2 θ and residual zincian malachite with peaks between 10 and 20 and 40 and 50 2 θ (high-temperature carbonate). Incomplete decomposition of the precipitate during calcination is essential for optimum catalyst performance.

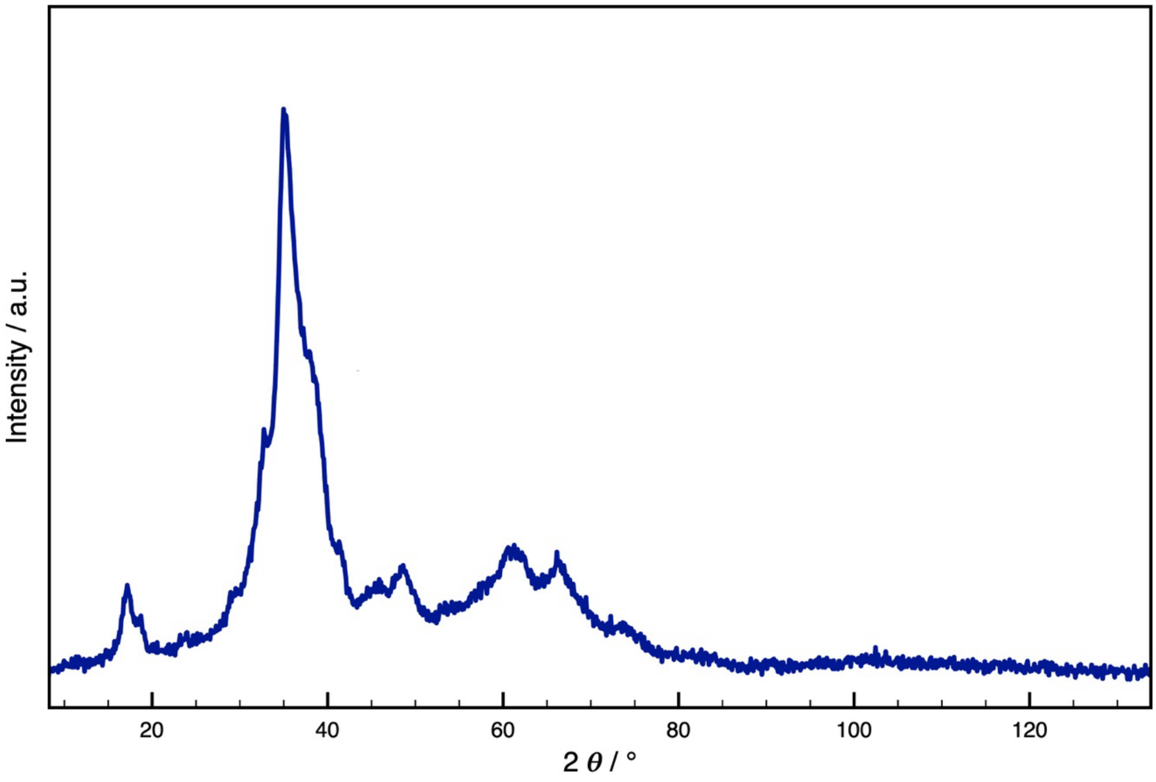

Figure S1. Powder X-ray diffraction pattern of CZA-prec.

Prior to the operando experiments, the powder was pressed and sieved and the sieve fraction (100−200 µm) of the pre-catalyst was activated using a reduction process at 250°C in 5 vol.% H_2 in N_2 with a flow rate of 0.7 ml min^{-1} for 90 min applying a heating rate of 2.4 K min^{-1}. The sample before activation is referred to as CZA-prec and after the activation process is referred to as CZA catalyst.

Thermogravimetry analysis coupled with a mass spectrometer (TGA/MS) was conducted using a NETZSCH STA 449C device for CZA-prec (FHI code: S35214).

Operando apparatus

The experimental apparatus containing a gas supply system, a stage for *operando* Microwave Cavity Perturbation Technique (MCPT) experiments and one for the *operando* EPR spectroscopy cell as well as an on-line gas chromatography (GC) analyzer is schematically depicted in Figure S2. The gas supply control system consists of various gas inlet lines with corresponding flow controllers (EL-FLOW Prestige, Bronkhorst), depicted as FC, which allow the required gas composition to be set. A pressure of 1.4 bar was maintained in the inlet gas reservoir using a pressure controller (P-702CV, Bronkhorst), shown as PC in the final feed line. A pneumatic selection valve (A66UWE, VICI) controlled by commercial software (iTool V9.79, Eurotherm) was used to direct the reactant gas stream to either the EPR or the MCPT experiments and the outlet gas stream to the GC. As thermocouples are metallic components, placing them in the center of a catalyst bed significantly disturbs the electromagnetic (EM) field in the cavity of both experiments. As a result, both MCPT and EPR *operando* cells have been designed so that the thermocouple remains outside the cavity, in contact with one side of the catalyst bed. The temperature within the catalyst bed was calibrated in separate experiments using a secondary K-type thermocouple placed it in the center of the catalyst bed for the EPR and MCPT cells, respectively.

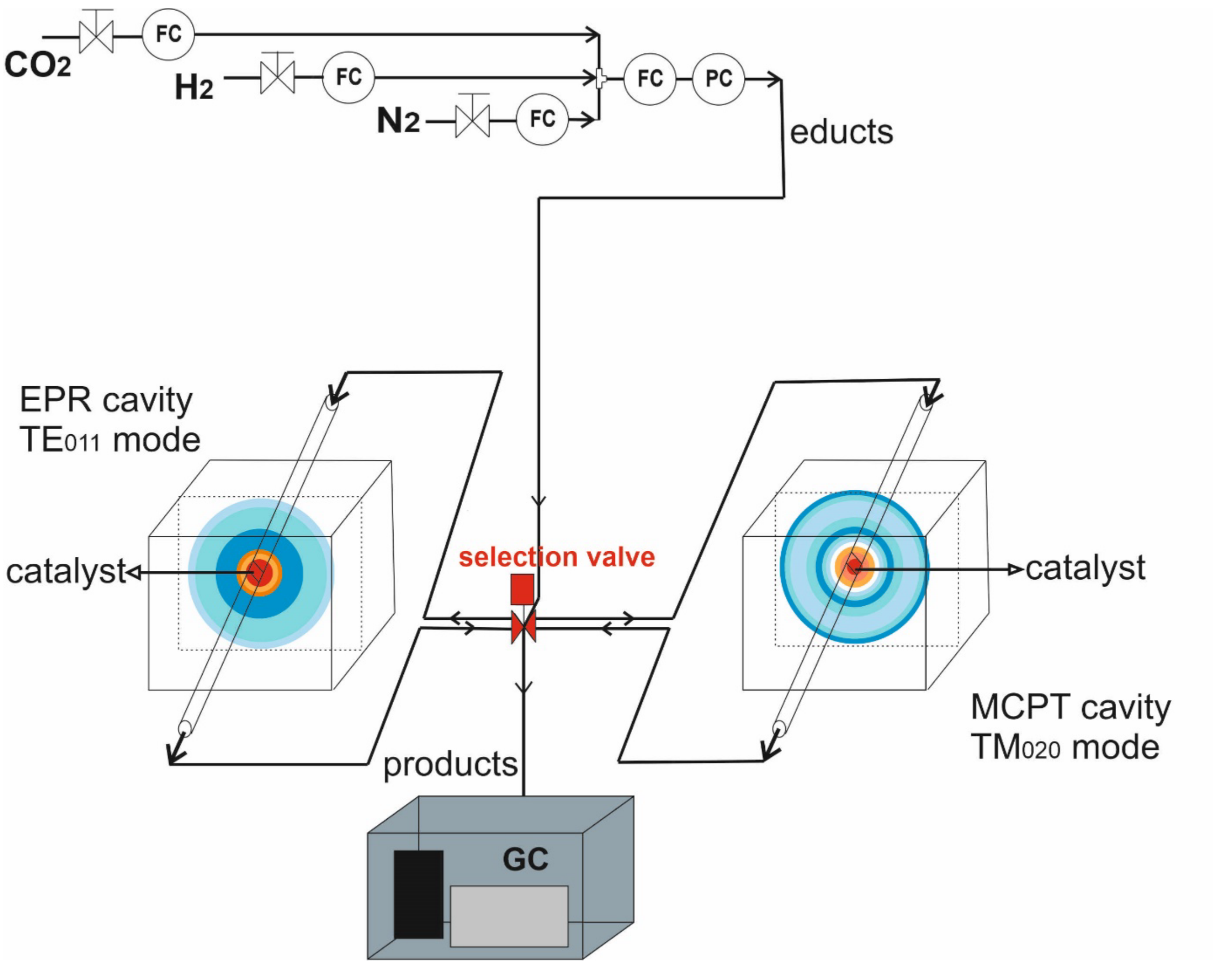

Figure S2. Schematic representation of the experimental apparatus including gas supply system, EPR and MCPT *operando* cells, GC device and selection valve to guide the inlet/outlet streams. The inlet feed and outlet product streams can either be guided through/from EPR or MCPT *operando* cells via a pneumatic selection valve.

Quality factor

In previous MCPT studies on Cu/ZnO based catalysts in r-WGS reaction, model catalysts with a low mass percentage of Cu were used to prevent larger changes in the Q-value of the resonator.[2] While a quantification of the changes in the Q-values in terms of changes in the conductivity is limited to small perturbations, the qualitative correlation between the changes of the Q-value and the changes in dielectric losses remains valid.[3] Therefore, *operando* MCPT measurements can be used to monitor changes in the dielectric losses even for samples with a high metallic content, such as for the present Cu/ZnO based catalyst, by recording the complex reflection coefficient ($\Gamma(\omega)$) and analyzing it in terms of the Q-value of the resonator. Equation 1 shows the definition of the complex reflection coefficient, which is a reflected voltage (V_-) divided by an incident voltage (V_+).[3] To visualize the process dependence of the complex reflection coefficient magnitude $|\Gamma(\omega)|$, the transition of the reflection mode, also

known as reflection loss (reflection magnitude versus frequency), before and after the activation processes is shown in Figure S3.

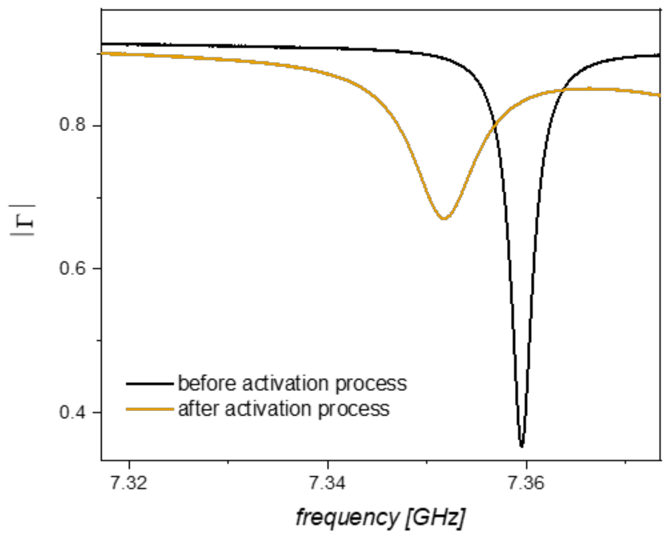

Figure S3. Changes of the reflection mode during the activation process. Black curve: at room temperature before activation, orange cure: at 250°C after the activation process.

The dimensionless quality factor (Q), conceptually described by equation 2, quantifies the ability of a resonator to store energy. The quality factor is influenced by the presence of microwave radiation absorbing material in the cavity, so this quantity provides a way to characterize the dielectric loss properties of a sample placed in the cavity.[4]

$$|\Gamma(\omega)| = \frac{V_-}{V_+} = \alpha + i\beta \quad (1)$$

$$Q = \frac{2\pi\ (maximum\ microwave\ energy\ stored\ in\ the\ resonator)}{energy\ dissipated\ by\ the\ resonator\ per\ microwave\ cycle} \quad (2)$$

Since the packing of a catalyst bed, its position and the total mass in the cavity can affect the Q-value, the CZA catalyst was not replaced with a new CZA-prec after one experiment, but re-activated without moving the catalyst bed in the reactor. Since all experiments were conducted using the same amount of catalyst, the effect of catalyst mass on the yield of CO or Q-values can be neglected in the results section.

MCPT measurements

The *operando* MCPT cell was elaborately described by Eichelbaum *et al.*[5] The MCPT measurements were performed using an Agilent PNA-LN5230C vector network analyzer (VNA) attached to a cylindrical silver and gold-plated resonator (radius of 34 mm and height of

20 mm, TM_{020} mode 7.1- 7.37 GHz) using a MW power attenuation of 5 dBm. Reflection curves (s. Figure S3) were averaged for one minute resulting in a corresponding temporal resolution of the MCPT data. The CZA-prec (23 mg) was settled in a fixed bed quartz reactor tube with a nominal inner diameter of 3 mm, using quartz wool (Carl Roth GmbH + Co.KG) to fix the sample. The reflection curves recorded by the VNA device were analyzed using the "yadg" Python package, which is based on Kajfez's Q0REFL program, a non-linear least-squares curve fitting of a tied smith chart[6] giving the dimensionless Q-values. As the Q-values were determined for the TM_{020} mode of the resonator the term Q_{020} would usually be used. As we have used the same mode throughout this study, the simplified term "Q"-value is used.
The experiments to study the dielectric loss properties of the catalyst through *operando* MCPT were designed by increasing the partial pressure of one component (CO_2 or H_2) in the feed stream, while keeping the other one constant. The reflection mode traces used to determine the Q-value were averaged to result in one trace per minute during the reaction. To facilitate comparative analysis of the variation in the Q-value throughout steps of increasing H_2 or CO_2 partial pressure, the Q-value in the corresponding activation step (5 vol.% H_2 in N_2 - 250°C) was considered as a reference point. In this context, the term ΔQ is defined as in equation 3. Q_{act} and Q_{rea} represent the mean values of the last five Q-values measured under activation and operational conditions, respectively.

$$\Delta Q = Q_{rea} - Q_{act} \qquad (3)$$

EPR measurements

An *operando* EPR cell was developed in this research based on previous well-established *in-situ/operando* MW absorption cells for investigations of powder catalysts at gas-solid interfaces.[5, 7] The EPR experiments were conducted with a Bruker EMXplus armed with a high temperature resonator, ER 4114 HT (TE011 mode, longitudinal electric field was zero). Continuous wave EPR spectra were recorded at X-band frequencies (approx. 9.15 GHz) with a microwave power of 2.00 mW, MW power attenuation of 20 dB, while applying a modulation frequency of 100 kHz and a modulation amplitude of 0.3 mT. As the activated catalyst exhibits rather high dielectric losses it is crucial to position the sample in the center of the resonator and avoid critical coupling difficulties by using a properly sized reactor. In this study a homemade quartz reactor with an inner diameter of 1 mm made of Suprasil® quartz (ilmasil PN, QSIL GmbH) was designed and employed.

The simulation of the EPR spectra at room temperature and before the activation process was carried out using MATLAB R2019b with the EasySpin 5.2.35 package.[8] The spectra were simulated considering electron Zeeman- and hyperfine-interactions using an effective spin Hamiltonian as shown in equation 4.

$$H = \beta_e \cdot B^T \cdot g \cdot \hat{S} + \hat{S}^T \cdot A \cdot \hat{I} \quad (4)$$

β_e denotes the Bohr magneton, B is the applied magnetic field vector, $\hat{S}$ the vector operator of the electron spin and g and A are the tensors to describe the anisotropic Zeeman- and hyperfine interaction, respectively.

Figure S4 shows the EPR spectrum of the CZA-prec at room temperature and the simulated spectrum assuming isolated Cu(II) sites (S=1/2 and I=3/2, both ^{63}Cu and ^{65}Cu isotopes were considered with their naturally abundance.) The line shape of the simulated spectrum was obtained using the following principal components of the g and A tensors: [2.08 2.07 2.36] and [50 50 400], respectively. The g-values obtained from the simulation align reasonably well with previous EPR studies of Cu containing catalysts.[9] The almost axially symmetric g-tensor components with all principal components being larger than the g-value of the free electron (g_e = 2.0023) and $g_{\parallel}$ (parallel g-value, g_{zz}) > $g_{\perp}$ (perpendicular g-value, $g_{xx} = g_{yy}$) is typically found for a distorted octahedral coordination of the isolated Cu(II) ions with the d_{x2-y2} orbital being energetically lowest lying.[10]

It is, however, clear from the comparison of the measured and simulated spectrum presented in Figure S4 that the experimental spectrum cannot be observed assuming an ensemble of isolated Cu(II) ions with comparable local geometries. The residual shown in Figure S4 (top trace) suggests that the observed EPR signal is not solely due to isolated Cu(II) ions. Given the large amount of Cu(II)O in the CZA-prec (68.1 wt.% CuO according to XRF), the broad residual may stem from magnetically coupled Cu(II)-ions.[4, 11]

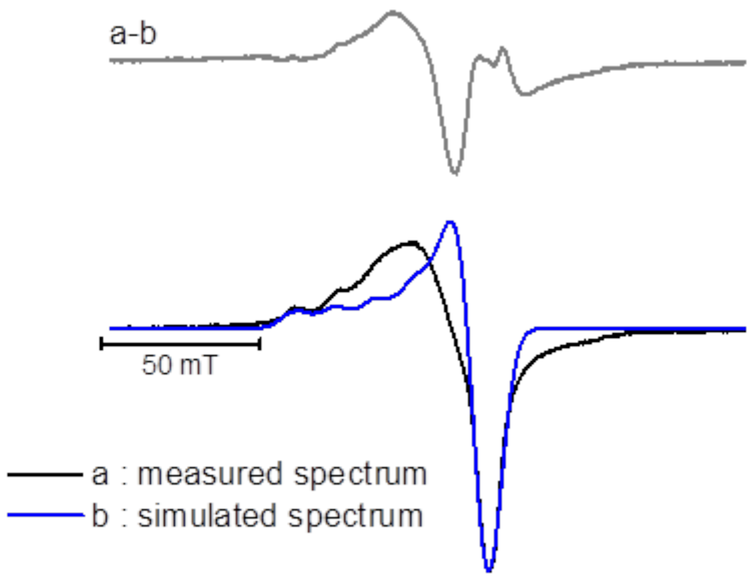

Figure S4. a) Base line corrected EPR spectrum of the CZA pre-catalyst at room temperature b) simulation spectrum of a. The upper curve shows the difference between the measured and simulated spectra.

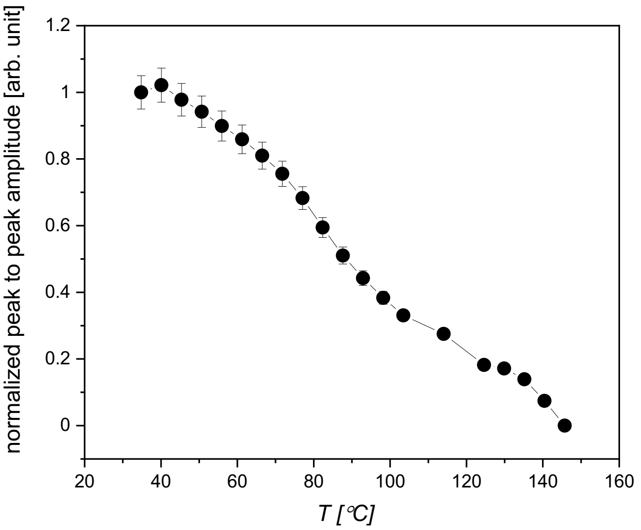

Figure S5. The decrease in amplitude of the EPR signal during activation of CZA-prec in 5 vol.% H_2 in N_2.

Product analysis and determination of kinetic parameters

The molar percentage of the produced CO was determined using an Agilent 7890A GC device equipped with a flame-ionization detector (FID) and a Polyarc® reactor (arc® activated research company), making a one-point calibration procedure more accurate.

Raw data of the determination of reaction orders are shown in Figure S6.

a)

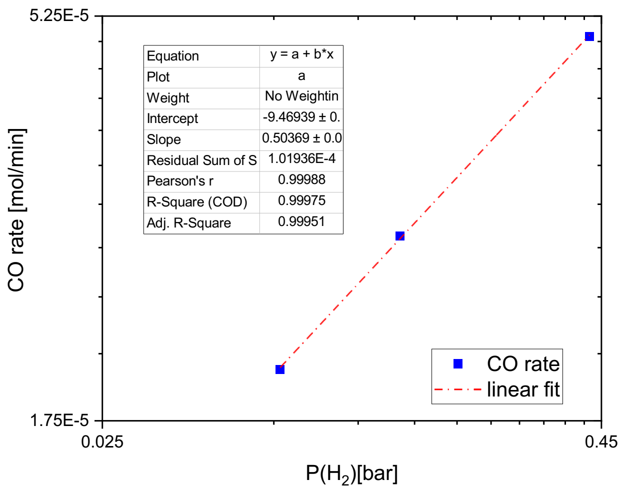

b)

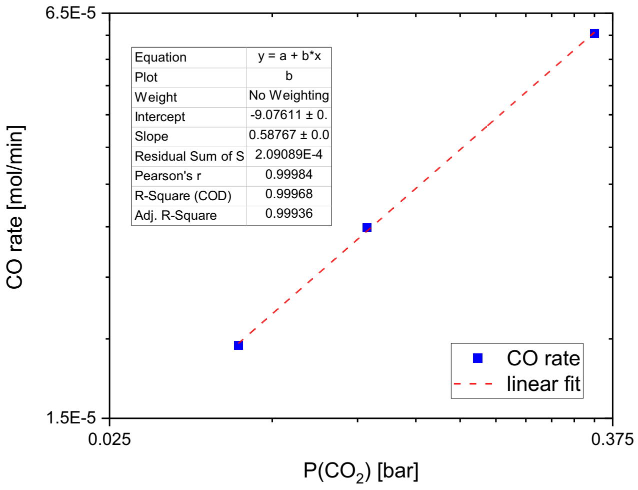

Figure S6. r-WGS reaction order towards a) H_2 and b) CO_2 at 230 ºC and a pressure of 1.4 bar using an activated CZA catalyst. Axes are logarithmic. Both partial pressure of CO_2 in section a) and H_2 is section b) was set to 100 mbar. The intersects in a) and b) show the statistical details of fitting.

2. Temporal evolution of Q-value and catalytic activity

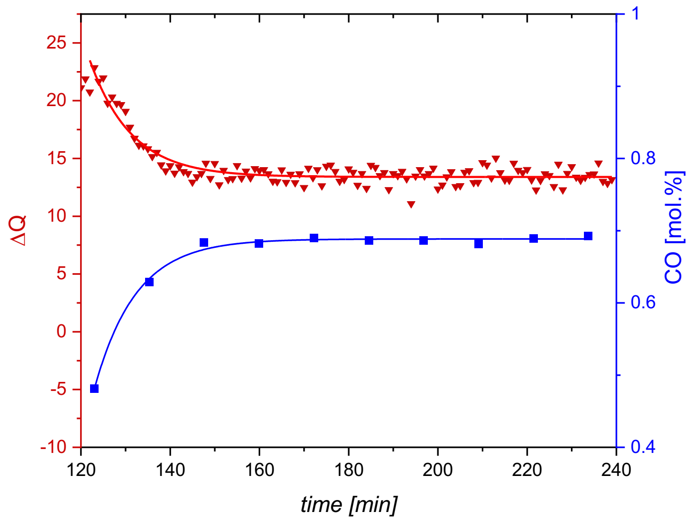

Figure S7. Exponential decay function fit of ΔQ and CO-yield. Data were taken from Figure 2b after changing the feed composition to a 1 : 1 ration of CO_2 : H_2. The solid lines are the fits to the data using a single exponential function and result in a time constant of 9 ± 1 min.

References

[1] J. Schumann, T. Lunkenbein, A. Tarasov, N. Thomas, R. Schlögl, M. Behrens, *ChemCatChem* **2014**, *6*, 2889.

[2] a) J. Schumann, M. Eichelbaum, T. Lunkenbein, N. Thomas, M. C. Alvarez Galvan, R. Schlögl, M. Behrens, *ACS Catal.* **2015**, *5*, 3260; b) E. H. Wolf, PhD thesis, Freie Universität Berlin (Berlin), **2020**.

[3] L. F. Chen, C. K. Ong, C. P. Neo, V. V. Varadan, V. Varadan, in *Microwave Electronics*, John Wiley & Sons, **2004**, pp. 37-141.

[4] J. Weil, A., J. R. Bolton, *Electron Paramagnetic Resonance: Elementary Theory and Practical Applications, Appendix F: Experimental Considerations*, second ed., Wiley, Hoboken, **2006**.

[5] M. Eichelbaum, R. Stößer, A. Karpov, C. K. Dobner, F. Rosowski, A. Trunschke, R. Schlögl, *Phys. Chem. Chem. Phys.* **2012**, *14*, 1302.

[6] P. Kraus, E. H. Wolf, C. Prinz, G. Bellini, A. Trunschke, R. Schlögl, *Digital Discovery* **2022**, *1*, 241-254.

[7] A. Brückner, P. Rybarczyk, H. Kosslick, G.-U. Wolf, M. Baerns, *Stud. Surf. Sci. Catal.* **2002**, *142*, 1141-1148.

[8] S. Stoll, A. Schweiger, *J. Magn. Reson.* **2006**, *178*, 42-55.

[9] a) J. Wen, C. Huang, Y. Sun, L. Liang, Y. Zhang, Y. Zhang, M. Fu, J. Wu, L. Chen, D. Ye, *Catalysts* **2020**, *10*; b) P. J. Carl, S. C. Larsen, *J. Catal.* **1999**, *182*, 208-218.

[10] E. Garribba, G. Micera. *J Chem Educ* **2006**, *83*, 1229-1232.

[11] H. Jacobsen, S. M. Gaw, A. J. Princep, E. Hamilton, S. Tóth, R. A. Ewings, M. Enderle, E. M. H. Wheeler, D. Prabhakaran, A. T. Boothroyd, *Phys. Rev. B Condens. Matter* **2018**, *97*.
